# Influence of menstrual cycle phase on resting-state functional connectivity in naturally cycling, cigarette-dependent women

**DOI:** 10.1186/s13293-016-0078-6

**Published:** 2016-05-10

**Authors:** Reagan R. Wetherill, Kanchana Jagannathan, Nathan Hager, Melanie Maron, Teresa R. Franklin

**Affiliations:** Department of Psychiatry, University of Pennsylvania, Philadelphia, PA 19104 USA

**Keywords:** Resting-state functional connectivity, Menstrual cycle, Neuroimaging

## Abstract

**Background:**

Sex differences in tobacco-related morbidity and mortality exist, with women experiencing more severe health consequences and greater difficulty with smoking cessation than men. One factor that likely contributes to these sex differences is menstrual cycle phase and associated neural and cognitive changes associated with ovarian hormone fluctuations across the menstrual cycle. Previously, we showed that naturally cycling, cigarette-dependent women in the follicular phase of their menstrual cycle showed greater reward-related neural activity and greater craving during smoking cue exposure. To better understand our results and the observed sex differences in smoking behavior and relapse, we explored potential menstrual cycle phase differences in resting-state functional connectivity (rsFC) in naturally cycling, cigarette-dependent women. Understanding how menstrual cycle phase affects neural processes, cognition, and behavior is a critical step in developing more efficacious treatments and in selecting the best treatment option based on a patient’s needs.

**Methods:**

Resting-state functional connectivity analyses were used to examine connectivity strength differences between naturally cycling, premenopausal, cigarette-dependent women who were in the follicular phase (FPs; *n* = 22) and those in the luteal phase (LPs, *n* = 16) of their menstrual cycle. We also explored associations between connectivity strength and attentional bias to smoking cues.

**Results:**

Compared with LPs, FPs showed decreased rsFC between the dorsal anterior cingulate cortex (dACC) and the subgenual anterior cingulate cortex, medial orbitofrontal cortex (mOFC), and ventral striatum. Among FPs, rsFC strength between the dACC and the bilateral dorsolateral prefrontal cortex (DLPFC), the bilateral dorsal striatum, and the left temporal gyrus was inversely correlated with attentional bias to smoking cues.

**Conclusions:**

This is the first study to explore menstrual cycle phase differences in rsFC among cigarette-dependent women, and results suggest that FPs show differences in rsFC underlying cognitive control, which could place them at greater risk for continued smoking and relapse. These findings provide new insights toward individualized treatment strategies.

**Electronic supplementary material:**

The online version of this article (doi:10.1186/s13293-016-0078-6) contains supplementary material, which is available to authorized users.

## Background

Cigarette smoking remains the leading cause of preventable death in the USA [1], and sex differences in tobacco-related morbidity and mortality exist. Specifically, women experience more severe health consequences from cigarette smoking, including a 25 % increased risk of developing coronary heart disease and chronic obstructive pulmonary disease [2], a higher incidence of lung cancer [3], and an increased incidence of lung cancer-related deaths [4]. Fortunately, smoking cessation at any age can reduce excess risk of tobacco-related diseases and death, yet women have greater difficulty with smoking cessation than men. Indeed, research suggests that women are less able to quit smoking than men, either when attempting to quit on their own [5] or with the aid of treatment [6–8]. Thus, understanding the factors that contribute to these sex differences is vital to improving women’s health and smoking cessation interventions.

Preclinical and clinical research suggests that ovarian hormones (i.e., estradiol and progesterone), which fluctuate over the course of the menstrual cycle [9], influence smoking behavior, and relapse vulnerability. For example, preclinical studies indicate that estradiol increases dopamine release in the ventral striatum [10, 11], contributes to faster acquisition of drug-seeking behavior and escalation of drug consumption, and accelerates drug-primed and drug-cue induced reinstatement, an animal model of relapse [12–14]. Preclinical studies of progesterone, however, indicate that high levels of progesterone decrease motivation for nicotine, as demonstrated by decreased nicotine self-administration [15]. Similarly, female smokers who were administered exogenous progesterone exhibited decreased positive subjective effects of cigarette smoking [16] and reduced urges to smoke [16, 17]. Collectively, these studies suggest that estradiol enhances reward and vulnerability to continued smoking and relapse in women; whereas, progesterone may protect against smoking-related behaviors and relapse [15, 18, 19], and as such, the fluctuation in ovarian hormones that occurs across the menstrual cycle may contribute to sex differences in smoking behavior and relapse.

Functional magnetic resonance imaging (fMRI) has become a powerful tool in elucidating neural features underlying behavioral and cognitive differences, including sex and menstrual cycle phase differences. Our research group recently examined whether neural responses to appetitive smoking cues differed between naturally cycling, cigarette-dependent women who were in the follicular phase (FPs; low progesterone to estradiol ratio) compared to cigarette-dependent women who were in the luteal phase (LPs; high progesterone to estradiol ratio) of their menstrual cycle [20]. Findings indicated that FPs showed greater smoking cue-elicited craving and greater neural responses to smoking cues in the medial orbitofrontal cortex (mOFC) compared with LPs. Current evidence suggests that the mOFC is critically involved in the representation of the affective value of reward-related cues and reward-related decision-making [21, 22]. Thus, one potential explanation for these findings is that women in the follicular phase of their menstrual cycle may have greater connectivity between the mOFC and other reward-related brain regions compared to when they are in the luteal phase, perhaps resulting in less top-down, cognitive control and greater responses to appetitive smoking cues, increased craving, and vulnerability to relapse during a quit attempt.

Although fMRI provides information on brain activity in discrete regions, rsFC approaches allow for an examination of functional interactions between brain regions by identifying synchronized spontaneous fluctuations in the blood oxygen level-dependent (BOLD) fMRI signal [23, 24] or regional cerebral blood flow (CBF) [25] in the absence of a goal-directed task, (i.e., at rest). Thus, rsFC is thought to represent inherent brain organization, which influences brain function [26] and behavior [27]. While the vast majority of resting-state studies use BOLD fMRI resting-state data to examine rsFC, arterial spin labeling (ASL) perfusion fMRI provides the ability to image sustained brain activity because of its long-term temporal stability and to noninvasively quantify CBF [28]. Indeed, research indicates that ASL perfusion MRI is comparable to BOLD fMRI in assessing rsFC and resting-state networks [29]. To date, four studies have examined the effects of menstrual cycle phase on rsFC and yielded inconsistent findings [30–33]. Using independent components analysis (ICA), Petersen and colleagues (2014) conducted a cross-sectional study of rsFC in naturally cycling women and women using combined oral contraceptive pills and found that follicular females showed increased rsFC between the anterior default mode network (i.e., bilateral superior medial gyri, bilateral cingulate cortex, bilateral angular gyri, bilateral inferior frontal gyri, bilateral temporal poles, cerebellar vermis, right parahippocampal gyrus, right insular lobe, and right caudate nucleus) and the left angular gyrus compared with the luteal group. Follicular females also showed increased connectivity with the executive control network (i.e., bilateral cingulate cortex, bilateral supramarginal gyri, left insular lobe, bilateral middle frontal gyri, and right cuneus) and the right anterior cingulate cortex compared with luteal females [31]. Significant menstrual cycle phase differences were also observed in a longitudinal rsFC analysis of a single subject across four menstrual cycles using eigenvector centrality mapping [32]. Arelin and colleagues (2015) found that high levels of progesterone (i.e., luteal phase) were associated with increased rsFC between the bilateral sensorimotor cortex, right dorsolateral prefrontal cortex (DLPFC), and hippocampus. Two other studies examining menstrual cycle phase effects on rsFC using ICA found no menstrual cycle effects [30, 33]. Although informative, these studies were conducted in nonsmokers and tobacco use was not assessed or accounted for in analyses, and as such, research into the potential menstrual cycle phase differences in rsFC in naturally cycling, cigarette-dependent women is warranted.

The goal of the present study is to improve our understanding of smoking-related sex differences, specifically differences in inherent brain connectivity that may increase or protect against vulnerability to smoking behaviors and relapse. To this end, we expand upon previous research [20, 34] by examining menstrual cycle phase differences in rsFC of the mOFC cluster that differed between FPs and LPs during smoking cue exposure. Based on our previous findings and research suggesting that the follicular phase (low progesterone to estradiol ratio) is associated with increased activation of reward-related circuitry [35], we hypothesized that FPs would exhibit increased functional connectivity between the mOFC and other reward-related brain regions. In order to explore potential luteal phase effects, we focused on rsFC of the dorsal anterior cingulate cortex (dACC), a brain region shown to be involved in cognitive control, craving reappraisal, and modulation of reactivity to cues [34, 36–38]. Given that the luteal phase is associated with higher levels of progesterone and protection against vulnerabilities to smoking behaviors and relapse, we hypothesized that LPs would show greater rsFC between the dACC and reward-related regions, as the dACC could be exerting more cognitive control over reward-related responses.

## Methods

### Participants

Participants were recruited via radio advertisements and local list-serves describing ongoing studies examining individual differences in smoking cue reactivity and smoking behavior [20, 39]. All eligible and interested participants provided informed consent prior to their inclusion in the study. Telephone screens and medical and psychiatric evaluations were used to assess participant eligibility during an in-person screening visit. Ineligible participants were those who reported other current substance dependence, had current Axis I *DSM-IV* psychiatric diagnoses, had significant medical conditions, reported a history of head trauma or injury causing loss of consciousness lasting greater than three minutes or associated with skull fracture or inter-cranial bleeding, or had irremovable magnetically active objects on or within their body. Males, pregnant or lactating women, post- or peri-menopausal women, women with irregular menstrual cycle length or outside the range of 26–30 days, women using exogenous hormones and/or hormonal contraceptives, and women currently experiencing difficulties with their menstrual cycle (e.g., spotting between menses, current diagnoses of premenstrual syndrome or premenstrual dysphoric disorder) were also excluded.

Thirty-eight physically healthy premenopausal smokers ranging in age from 21 to 51 years were included in the current analyses (Table 1). Perfusion fMRI data from these participants were previously reported in a study examining the influence of menstrual cycle phase on neural responses to smoking cues [20]. Participants received $100.00 for completing the study. The study adhered to the Declaration of Helsinki and the University of Pennsylvania Institutional Review Board approved all procedures.

**Table 1:** Participant characteristics

	All *N* = 38	Follicular phase *n* = 22	Luteal phase *n* = 16	*p*
Demographics				
Age	33.9 ± 1.7	32.4 ± 2.3	35.9 ± 2.5	0.31
African American (*n*), %	50.0 (19)	50.0 (11)	50.0 (8)	1.00
Years of education	14.4 ± 0.3	14.4 ± 1.8	14.5 ± 2.6	0.90
Smoking characteristics				
Cigarettes per day	12.6 ± 0.7	11.5 ± 0.9	13.9 ± 1.1	0.12
Years smoking	14.6 ± 1.8	12.7 ± 2.4	17.3 ± 2.5	0.21
Pack years^a^	10.3 ± 1.5	8.6 ± 2.0	12.7 ± 2.3	0.20
FTND scores	4.4 ± 0.3	4.1 ± 0.3	4.9 ± 0.4	0.13

*FTND* Fagerström test for nicotine dependence, *FTND* scores ranged from 1 to 9

^a^Pack years calculation: cigarettes per day (÷) cigarettes in a pack (×) years smoking

### Measures

During the screening visit, participants completed the Mini International Neuropsychiatric Interview [40], which assessed current *DSM-IV* diagnosis of substance dependence other than nicotine and current severe psychiatric symptoms. Participants also completed the Fagerström Test for Nicotine Dependence (FTND) [41], which was used to determine severity of nicotine dependence, and the Menstrual Cycle Questionnaire (MCQ), a measure developed within our laboratory that assesses menstrual cycle information and was used to identify menstrual cycle phase. The MCQ provides information on menstrual cycle length, regularity, first day of last menses, premenstrual symptoms, and method of birth control (Additional file 1). A brief MCQ is administered on the day of scanning and is used to determine what day of the menstrual cycle the participant is on when scanning occurs. Based on this information, women were either excluded or separated into two groups: follicular phase females or luteal phase females. The luteal phase was defined as the 8 days prior to the first day of menses, and the follicular phase comprised the first 7 days after the onset of menses [42, 43]. Menstrual cycle data was collected over one to two menstrual cycles and depended on when the participant completed their scanning session.

### Attentional bias to smoking cues

On the day of the neuroimaging scanning session, 34 participants (19 FPs) completed an off-magnet visual dot-probe behavioral task, described in detail elsewhere [44, 45], designed to objectively measure attention shifts toward smoking pictures. Briefly, participants were sated when completing the task, which consisted of 20 color photographs of smoking-related content (e.g., pack of cigarettes) and 20 photographs not specifically related to smoking (e.g., pack of playing cards). Participants were told that picture pairs would briefly flash on the screen followed by an asterisk (the dot probe) in the position previously occupied by one of the pictures. Participants were asked to indicate the position of the target as quickly and accurately as possible by using the left and right index fingers to strike the left or right response key, respectively. After a 20-trial practice block containing picture pairs without smoking-related stimuli, participants completed 80 stimulus pair trials. Each stimulus pair was presented four times, counterbalancing for picture/dot-probe location. For each subject, a relative attentional bias score was computed as RT_non-smoking_ − RT_smoking_ with positive bias scores reflecting attentional bias for SCs and negative scores reflecting bias toward non-smoking cues.

### Resting-state scan

*Pseudo*-continuous arterial spin-labeled (*p*CASL) perfusion fMRI, a quantitative estimate of CBF and indirect measurement of neural activity [46], measured resting-state CBF. Before the scanning session, participants smoked a cigarette to satiety in the presence of study personnel to minimize nicotine withdrawal-induced craving that might accrue during the scanning session. The 5-min *p*CASL resting-state scan was acquired first in a 40-min scanning session to avoid task-related cerebral blood flow influences. The scan was acquired approximately 20–25 min after smoking to allow the acute cardiovascular effects of smoking to dissipate. Prior to the resting-state scan, participants are provided the following instructions: “Please try not to move your head or any other part of your body. Relax and clear your mind as much as you can. Remember to stay awake and keep your eyes open.”

### Imaging approach and acquisition

Imaging data were acquired on a 3.0 Tesla Trio whole-body scanner (Siemens AG, Erlangen, Germany). For co-registration of the functional data, a T1-weighted three-dimensional (3D) high resolution magnetization-prepared rapid acquisition gradient echo (MPRAGE) scan was acquired with repetition time (TR) = 1620 ms, echo time (TE) = 3.09 ms, inversion time (TI) = 960 ms, 192 × 256 × 160 matrix, slice thickness = 1 mm, and flip angle = 15° for 32 subjects and TR = 1620 ms, TE = 3.87 ms, TI = 950 ms, 192 × 256 × 160 matrix, slice thickness = 1 mm, and flip angle = 15° for six subjects. *p*CASL perfusion fMRI sequence was used for resting-state data acquisition. Interleaved images with and without labeling were obtained using a gradient echo echo-planar imaging sequence with a post-labeling delay of 1000 ms, field of view (FOV) = 220 × 220 mm^2^, 64 × 64 matrix, TR/TE = 3500/17 ms, flip angle = 90°, slice thickness = 6 mm, voxel size = 3.438 × 3.428 × 7.2 mm with a 2-mm inter-slice gap for 32 subjects and post-labeling delay of 700 ms, FOV = 220 × 220 mm^2^, 64 × 64 matrix, TR/TE = 3000/17 ms, flip angle = 90°, slice thickness = 8 mm, voxel size = 3.438 × 3.428 × 10 mm with a 1.2 mm inter-slice gap for six subjects. A homogeneity of variance test assessed whether data acquisition differences influenced findings, and no significant differences were observed.

### Statistical analyses

A Statistical Parametric Mapping (SPM)-based arterial spin labeling (ASL) data processing toolbox [47] was used for *p*CASL perfusion data analyses, as described previously [48, 49]. Briefly, the first step in our pre-processing pipeline was motion correction (MoCo) and denoising [50]. Next, ASL image pairs were realigned to the mean of all control images and spatially smoothed with a 3D isotropic Gaussian kernel at 9-mm full width at half maximum (FWHM). For resting-state data, 38 CBF image series were generated from the 76 label/control ASL image pairs using the same methods for CBF calculations. The mean control image of each subject’s data was co-registered to the structural image using the mutual information based co-registration algorithm provided by SPM8. The same transformation parameters were applied to co-register the CBF maps to each subject’s anatomical image. Subsequently, the structural image was spatially normalized to the Montreal Neurological Institute (MNI) standard brain. The resulting transformation matrix was used to align the CBF images to MNI space. A binary brain mask was used to exclude the non-brain areas in the CBF maps.

Correlation analyses examined menstrual cycle phase differences in the temporal relationship between the mOFC seed region and other brain regions and between the dACC seed region and other brain regions. The functionally identified, mOFC seed region (10-mm sphere centered at [−2, 38, −20]) was used based on our previous study showing menstrual cycle phase differences in neural responses to smoking cues [20]. We centered another 10-mm sphere seed in the dACC [0, 10, 38] to explore potential modulation of reward circuitry. A cross-correlation coefficient (CC) map was obtained for each seed by correlating the average time course of the seed region with each voxel’s time course over the brain. The resulting correlation coefficients were converted to *z*-scores using Fisher’s *r*-to-*z* transformation. The Z maps were then analyzed in a random-effects model in SPM8 to compare FP and LP connectivity. We identified regions showing differences in connectivity strength with a significant voxelwise statistical threshold (*p* < 0.005), and to control for multiple comparisons, voxels were required to be part of a cluster >285 voxels, as determined by a Monte-Carlo simulation using the corrected version of *3dClustSim* (Analysis of Functional NeuroImages, https://afni.nimh.nih.gov/) that resulted in 5 % probability (corrected) of a cluster surviving due to chance.

Secondary analyses. Secondary regression analyses examined potential associations between rsFC of our two a priori seed regions (i.e., mOFC and dACC) and attentional bias to smoking cues scores, a clinically significant measure of implicit processing and selective attention for smoking-related stimuli [51]. Separate regression analyses were conducted for FPs (*n* = 19) and LPs (*n* = 15). Sample sizes for the secondary analyses decreased due to missing and/or unusable attentional bias data. The statistical threshold for the exploratory secondary analyses was *p* < 0.01 with voxels required to be part of a cluster >100 voxels.

### Demographic and behavioral statistical analyses

Continuous demographic variables were summarized by calculating means and standard error measurements (X ± SEMs). Independent samples *t* tests compared follicular and luteal females on continuous variables. Nominal demographic variables were summarized by calculating proportions and compared across groups using chi-square analyses.

## Results

### Participant characteristics

Table 1 provides participant demographics and smoking characteristics. There were no significant menstrual cycle phase differences in age, education, race/ethnicity, cigarettes per day, number of years smoking, pack years, or FTND scores (*p*s > 0.12).

### Resting-state functional connectivity analyses

**Fig. 1:**
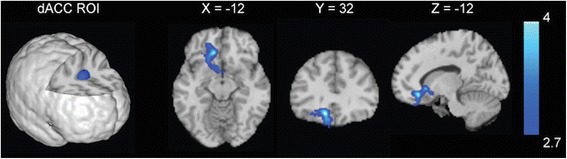
Menstrual cycle phase differences in resting-state functional connectivity (rsFC) strength. Compared with cigarette-dependent women in the luteal phase of their menstrual cycle (LPs), cigarette-dependent women in the follicular phase of their menstrual cycle (FPs) showed decreased rsFC strength between the dorsal anterior cingulate cortex (dACC) seed region and a large cluster spanning the mOFC, subgenual anterior cingulate cortex, and the striatum. Images are displayed neurologically (left is left)

Analyses examining menstrual cycle phase differences in resting-state connectivity in cigarette-dependent women revealed no significant differences in rsFC of the mOFC between FPs and LPs. For the dACC, FPs showed decreased rsFC between the dACC and a large cluster spanning the left subgenual anterior cingulate cortex (sgACC), mOFC, and ventral striatum compared with LPs (Fig. 1). FPs also exhibited increased connectivity strength between the dACC and a cluster in the supplementary motor area/precentral gyrus compared with LPs.

#### Secondary analyses

**Fig. 2:**
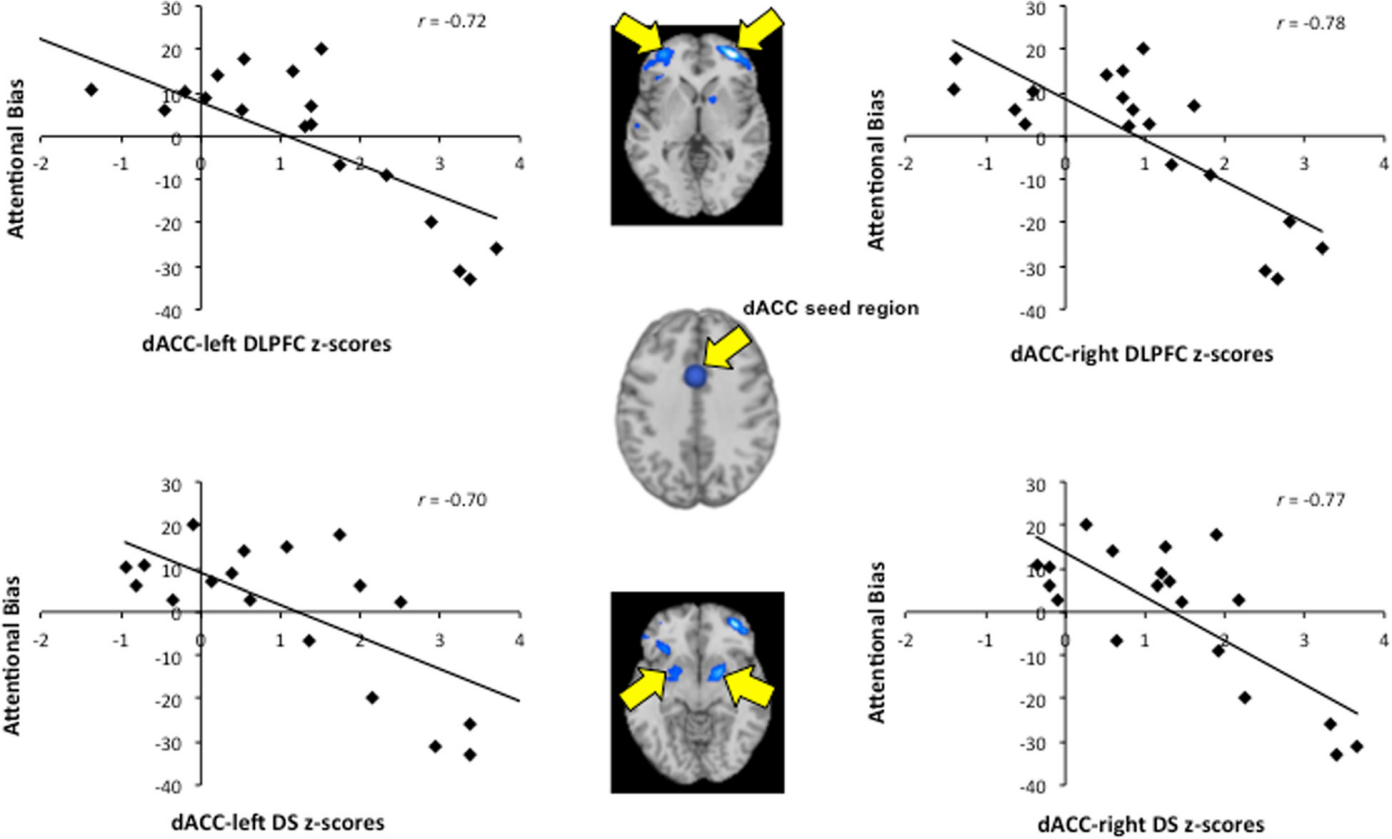
Associations between dorsal anterior cingulate cortex (dACC) resting-state functional connectivity (rsFC) and attentional bias to smoking cue scores within the cigarette-dependent women in the follicular phase of their menstrual cycle (FPs) group. The *plots* show the correlations between the dACC seeded rsFC strength and attentional bias to smoking cue scores acquired when FPs were sated and prior to the scanning session. *Values on the axes* are the z-scores extracted from the secondary analyses exploring the association between dACC rsFC strength and attentional bias to smoking cues at a threshold of *p* < .01 and clusters >100 voxels. Images are displayed neurologically (left is left). *DLPFC* dorsolateral prefrontal cortex, *DS* dorsal striatum

In an attempt to shed light on potential mechanisms underlying menstrual cycle phase differences, exploratory secondary analyses examined associations between rsFC and attentional bias to smoking cues. Among FPs, rsFC strength between the dACC and the bilateral dorsolateral prefrontal cortex (DLPFC), the bilateral dorsal striatum, and the left temporal gyrus showed inverse correlations with attentional bias to smoking cue scores (Fig. 2). There were no other significant correlations between rsFC of the mOFC or the dACC and attentional bias scores among FPs or LPs.

## Discussion

To our knowledge, this is the first study to examine menstrual cycle phase effects on rsFC in cigarette-dependent women. Findings revealed unique menstrual cycle phase differences in dACC rsFC. Specifically, FPs exhibited decreased rsFC strength between the dACC and reward-related regions compared with LPs. Exploratory secondary analyses showed that rsFC between the dACC and reward-related regions was inversely correlated with attentional bias to smoking cues among FPs. These findings provide new insights into menstrual cycle effects on rsFC and potential mechanisms underlying sex differences in smoking behavior and relapse.

Contrary to expectations, FPs and LPs did not show differences in mOFC rsFC at our conservative statistical thresholds. When we relaxed statistical thresholds to *p* < 0.01, we observed increased connectivity strength between the mOFC and caudal DLPFC in FPs compared with LPs, which is similar to findings reported by Petersen and colleagues (2014) suggesting that women in the follicular phase show increased connectivity of the anterior default mode network (DMN) compared with women in the luteal phase [31]. Given our small sample size and cross-sectional approach, future research in a larger, longitudinal sample of women could provide more insight into potential differences in mOFC rsFC of women in their follicular phase compared with their luteal phase.

In previous research, Hong and colleagues (2010) found that smokers exhibited reduced rsFC strength between the dACC and the ventral striatum and posited that weakened connectivity strength within this circuit represents a “trait-like” biomarker of nicotine addiction [52]. Here, we replicated these earlier findings of altered functional connectivity strength between the dACC and the ventral striatum and extended findings to include other reward-related regions. Structurally, the dACC projects to the entire striatum [53], and functionally, studies suggest that the dACC is involved in reward-related decision-making [54, 55], conflict monitoring [56, 57], and craving regulation in smokers [34]. Thus, decreased connectivity strength between the dACC and reward-related regions in FPs may reflect reduced cognitive regulation over smoking behaviors.

Although participants in this study were cigarette-dependent, the seed regions chosen for analyses are not exclusively related to smoking or smoking behaviors, and all participants were sated (not experiencing nicotine withdrawal) at the time of scanning. Consequently, our findings may represent a more fundamental effect of menstrual cycle phase on rsFC rather than an effect observed only in cigarette-dependent women. Thus, findings could suggest that women in the follicular phase of their menstrual cycle may show similar decreases in rsFC between the dACC and reward-related regions and experience reduced cognitive control over rewarding behaviors in general. This interpretation is speculative, and additional research into this possibility is warranted.

With repeated cigarette use and associated dopamine-related processes within the mesocorticolimbic and nigrostriatal circuits, smoking-related cues acquire incentive-motivational salience, which gives them the ability to trigger craving [58]. Smoking cues also acquire attentional salience, which manifests as an attentional bias for smoking cues in cigarette-dependent individuals [45, 51]. Consequently, we explored the relationship between resting-state connectivity and attentional bias to smoking cues in an attempt to identify potential mechanisms underlying the observed menstrual cycle phase differences and menstrual cycle effects on smoking-related behaviors. We found that attentional bias to smoking cues among FPs was inversely correlated with rsFC strength between the dACC and the bilateral DLPFC, the bilateral dorsal striatum, and a cluster in the left temporal gyrus. Thus, the decreased connectivity observed among FPs may reflect or partially reflect dACC dysfunction that could result in greater attentional bias to smoking cues and associated reward-related responses to smoking cues [20]. Based on this secondary analysis, we speculate that altered dACC connectivity during the follicular phase of the menstrual cycle may serve as a neural substrate of reduced cognitive regulation over smoking-related cognition and behaviors.

We previously showed that FPs had greater smoking cue-elicited craving and greater responses in the mOFC when viewing 10-min video clips of appetitive smoking reminders (compared to clips of non-smoking material) [20]. In the present study, we tested whether menstrual cycle phase influences rsFC in cigarette-dependent female smokers in order to better understand our results and the observed sex differences in smoking behavior and relapse [5–8]. Understanding how menstrual cycle phase affects neural processes, cognition, and behavior is a critical step in developing more efficacious treatments and in selecting the best treatment option based on a patient’s needs. Given that FPs exhibited altered rsFC between cognitive control and reward-related regions compared with LPs, we posit that decreased dACC-sgACC/mOFC/striatal connectivity strength may reflect impaired regulatory processes that could contribute to continued smoking behavior and increased relapse vulnerability among female smokers in the follicular phase of their menstrual cycle. As such, our findings suggest that women who opt to make a quit attempt during their follicular phase may benefit from additional support and treatment approaches that focus on increasing cognitive control, such as cognitive bias modification [59, 60]. Further, findings support previous research indicating that female smokers may be more successful at smoking cessation if they set a quit date during their luteal phase [61, 62], when female smokers appear to have greater cognitive control over smoking-related urges and behaviors.

The current findings should be interpreted in light of the following limitations. Importantly, menstrual cycle phase was not biochemically verified, and thus, we used menstrual cycle phase as a proxy for ovarian hormone function. Although others have demonstrated that paradigmatic measures of verification align with menstrual cycle phase self-report [63, 64], knowledge of menstrual phase does not necessarily translate to the hormonal milieu. For example, over one third of menstrual cycles are anovulatory, and hormones do not fluctuate normally during anovulatory cycles [65]. Consequently, even if menstrual cycle phase was correctly identified in the current study, one must be cautious in extrapolating knowledge of menstrual cycle phase to knowledge of hormonal status. Indeed, we acknowledge that the most scientifically rigorous test of our hypothesis would be to acquire data within women at multiple time points across the menstrual cycle (biochemically verified). This study is also limited in that we focused solely on menstrual cycle differences in rsFC of the mOFC and dACC. We recognize that many factors and other resting-state and task-related networks are at play, including negative affect/mood, stress, and variance in genes [49, 66, 67]. As such, future research could explore these factors, as well as other cognitive constructs associated with cigarette use to help better understand how menstrual cycle phase influences smoking behavior and relapse vulnerability and how these factors contribute to sex differences.

## Conclusions

To our knowledge, the current data represent the first examination of menstrual cycle effects on rsFC in cigarette-dependent women. These findings, along with our previous research demonstrating greater neural responses to smoking cues and cue-elicited craving among FPs [20], suggest that cigarette-dependent women in the follicular phase of their menstrual cycle experience task-related functional and rsFC abnormalities that may place them at greater risk for continued smoking behavior and relapse should they make a quit attempt. As such, this study provides important information that may help guide individualized treatment strategies and improve smoking cessation rates.

## Additional file

Additional file 1: Menstrual Cycle Questionnaire. The menstrual cycle questionnaire (MCQ) used in this study. (DOCX 32 kb)

## Abbreviations

3D: three-dimensional
ASL: arterial spin labeling
BOLD: blood oxygen level-dependent
CBF: cerebral blood flow
CC: cross-correlation coefficient
dACC: dorsal anterior cingulate cortex
DLPFC: dorsolateral prefrontal cortex
DMN: default mode network
DS: dorsal striatum
DSM-IV: Diagnostic and Statistical Manual of Mental Disorders (Fourth Edition)
fMRI: functional magnetic resonance imaging
FOV: field of view
FPs: cigarette-dependent women in the follicular phase of their menstrual cycle
FTND: Fagerström Test for Nicotine Dependence
FWHM: full width at half maximum
ICA: independent components analysis
LPs: cigarette-dependent women in the luteal phase of their menstrual cycle
MCQ: Menstrual Cycle Questionnaire
MNI: Montreal Neurological Institute
MoCO: motion correction
mOFC: medial orbitofrontal cortex
MPRAGE: magnetization-prepared rapid gradient echo
pCASL: pseudo-continuous arterial spin labeling
rsFC: resting-state functional connectivity
SEM: standard error of the mean
sgACC: subgenual anterior cingulate cortex
SPM: Statistical Parametric Mapping
TE: echo time
TI: inversion time
TR: repetition time

## References

[1] United States Department of Health and Human Services (2014). The health consequences of smoking: 50 years of progress. A report of the surgeon general.

[2] Huxley RR, Woodward M (2011). Cigarette smoking as a risk factor for coronary heart disease in women compared with men: a systematic review and meta-analysis of prospective cohort studies. Lancet.

[3] Freedman ND, Leitzmann MF, Hollenbeck AR, Schatzkin A, Abnet CC (2008). Cigarette smoking and subsequent risk of lung cancer in men and women: analysis of a prospective cohort study. Lancet Oncol.

[4] Patel JD, Bach PB, Kris MG (2004). Lung cancer in US women: a contemporary epidemic. JAMA.

[5] Ward KD, Klesges RC, Zbikowski SM, Bliss RE, Garvey AJ (1997). Gender differences in the outcome of an unaided smoking cessation attempt. Addict Behav.

[6] Wetter DW, Kenford SL, Smith SS, Fiore MC, Jorenby DE, Baker TB (1999). Gender differences in smoking cessation. J Consult Clin Psychol.

[7] Scharf D, Shiffman S (2004). Are there gender differences in smoking cessation, with and without bupropion? Pooled- and meta-analyses of clinical trials of Bupropion SR. Addiction.

[8] Perkins KA, Scott J (2008). Sex differences in long-term smoking cessation rates due to nicotine patch. Nicotine Tob Res.

[9] Yen SSC, Jaffee RB, Barbieri RL (1999). Reproductive endocrinology: physiology, pathophysiology and clinical management.

[10] Di Paolo T, Rouillard C, Bedard P (1985). 17 beta-estradiol at a physiological dose acutely increases dopamine turnover in rat brain. Eur J Pharmacol.

[11] Thompson TL, Moss RL (1994). Estrogen regulation of dopamine release in the nucleus accumbens: genomic- and nongenomic-mediated effects. J Neurochem.

[12] Chen H, Matta SG, Sharp BM (2007). Acquisition of nicotine self-administration in adolescent rats given prolonged access to the drug. Neuropsychopharmacology.

[13] Becker JB, Hu M (2008). Sex differences in drug abuse. Front Neuroendocrinol.

[14] Carroll ME, Anker JJ, Perry JL (2009). Modeling risk factors for nicotine and other drug abuse in the preclinical laboratory. Drug Alcohol Depend.

[15] Lynch WJ, Sofuoglu M (2010). Role of progesterone in nicotine addiction: evidence from initiation to relapse. Exp Clin Psychopharmacol.

[16] Sofuoglu M, Babb DA, Hatsukami DK (2001). Progesterone treatment during the early follicular phase of the menstrual cycle: effects on smoking behavior in women. Pharmacol Biochem Behav.

[17] Sofuoglu M, Mitchell E, Mooney M (2009). Progesterone effects on subjective and physiological responses to intravenous nicotine in male and female smokers. Hum Psychopharmacol.

[18] Carroll ME, Anker JJ (2010). Sex differences and ovarian hormones in animal models of drug dependence. Horm Behav.

[19] Hudson A, Stamp JA (2011). Ovarian hormones and propensity to drug relapse: a review. Neurosci Biobehav Rev.

[20] Franklin TR, Jagannathan K, Wetherill RR, Johnson B, Kelly S, Langguth J (2015). Influence of menstrual cycle phase on neural and craving responses to appetitive smoking cues in naturally cycling females. Nicotine Tob Res.

[21] Kringelbach ML, Rolls ET (2004). The functional neuroanatomy of the human orbitofrontal cortex: evidence from neuroimaging and neuropsychology. Prog Neurobiol.

[22] Rolls ET (2004). The functions of the orbitofrontal cortex. Brain Cogn.

[23] Biswal B, Yetkin FZ, Haughton VM, Hyde JS (1995). Functional connectivity in the motor cortex of resting human brain using echo-planar MRI. Magn Reson Med.

[24] Fox MD, Snyder AZ, Vincent JL, Corbetta M, Van Essen DC, Raichle ME (2005). The human brain is intrinsically organized into dynamic, anticorrelated functional networks. Proc Natl Acad Sci U S A.

[25] Zou Q, Long X, Zuo X, Yan C, Zhu C, Yang Y (2009). Functional connectivity between the thalamus and visual cortex under eyes closed and eyes open conditions: a resting-state fMRI study. Hum Brain Mapp.

[26] Smith SM, Fox PT, Miller KL, Glahn DC, Fox PM, Mackay CE (2009). Correspondence of the brain’s functional architecture during activation and rest. Proc Natl Acad Sci U S A.

[27] Kelly AM, Uddin LQ, Biswal BB, Castellanos FX, Milham MP (2008). Competition between functional brain networks mediates behavioral variability. Neuroimage.

[28] Rao H, Wang J, Tang K, Pan W, Detre JA (2007). Imaging brain activity during natural vision using CASL perfusion fMRI. Hum Brain Mapp.

[29] Zhu S, Fang Z, Hu S, Wang Z, Rao H (2013). Resting state brain function analysis using concurrent BOLD in ASL perfusion fMRI. PLoS One.

[30] Hjelmervik H, Hausmann M, Osnes B, Westerhausen R, Specht K (2014). Resting states are resting traits—an FMRI study of sex differences and menstrual cycle effects in resting state cognitive control networks. PLoS One.

[31] Petersen N, Kilpatrick LA, Goharzad A, Cahill L (2014). Oral contraceptive pill use and menstrual cycle phase are associated with altered resting state functional connectivity. Neuroimage.

[32] Arelin K, Mueller K, Barth C, Rekkas PV, Kratzsch J, Burmann I (2015). Progesterone mediates brain functional connectivity changes during the menstrual cycle-a pilot resting state MRI study. Front Neurosci.

[33] De Bondt T, Smeets D, Pullens P, Van Hecke W, Jacquemyn Y, Parizel PM. Stability of resting state networks in the female brain during hormonal changes and their relation to premenstrual symptoms*.* Brain Res. 2015;1624:275–85. 10.1016/j.brainres.2015.07.045 26253822

[34] Zhao LY, Tian J, Wang W, Qin W, Shi J, Li Q (2012). The role of dorsal anterior cingulate cortex in the regulation of craving by reappraisal in smokers. PLoS One.

[35] Dreher JC, Schmidt PJ, Kohn P, Furman D, Rubinow D, Berman KF (2007). Menstrual cycle phase modulates reward-related neural function in women. Proc Natl Acad Sci U S A.

[36] Weissman DH, Gopalakrishnan A, Hazlett CJ, Woldorff MG (2005). Dorsal anterior cingulate cortex resolves conflict from distracting stimuli by boosting attention toward relevant events. Cereb Cortex.

[37] Motzkin JC, Baskin-Sommers A, Newman JP, Kiehl KA, Koenigs M (2014). Neural correlates of substance abuse: reduced functional connectivity between areas underlying reward and cognitive control. Hum Brain Mapp.

[38] Janes AC, Farmer S, Peechatka AL, Frederick Bde B, Lukas SE (2015). Insula-dorsal anterior cingulate cortex coupling is associated with enhanced brain reactivity to smoking cues. Neuropsychopharmacology.

[39] Wetherill RR, Jagannathan K, Shin J, Franklin TR (2014). Sex differences in resting state neural networks of nicotine-dependent cigarette smokers. Addict Behav.

[40] Sheehan B, Lecrubier Y, Sheehan K (1998). The Mini International Neuropsychiatric Interview (MINI): the development and validation of structured diagnostic interview for DSM-IV and ICD-10. J Clin Psychiatry.

[41] Fagerström KO, Schneider NG (1989). Measuring nicotine dependence: a review of the Fagerström Tolerance Questionnaire. J Behav Med.

[42] Lenton EA, Lawrence GF, Coleman RA, Cooke ID (1983). Individual variation in gonadotrophin and steroid concentrations and in the lengths of the follicular and luteal phases in women with regular menstrual cycles. Clin Reprod Fertil.

[43] Wilcox AJ, Dunson D, Baird DD (2000). The timing of the “fertile window” in the menstrual cycle: day specific estimates from a prospective study. BMJ.

[44] Ehrman RN, Robbins SJ, Bromwell MA, Lankford ME, Monterosso JR, O'Brien CP (2002). Comparing attentional bias to smoking cues in current smokers, former smokers, and non-smokers using a dot-probe task. Drug Alcohol Depend.

[45] Wetherill RR, Jagannathan K, Lohoff FW, Ehrman R, O'Brien CP, Childress AR (2014). Neural correlates of attentional bias for smoking cues: modulation by variance in the dopamine transporter gene. Addict Biol.

[46] Floyd TF, Ratcliffe SJ, Wang J, Resch B, Detre JA (2003). Precision of the CASL-perfusion MRI technique for the measurement of cerebral blood flow in whole brain and vascular territories. J Magn Reson Imaging.

[47] Wang Z, Aguirre GK, Rao H, Wang J, Fernandez-Seara MA, Childress AR (2008). Empirical optimization of ASL data analysis using an ASL data processing toolbox: ASLtbx. Magn Reson Imaging.

[48] Franklin TR, Lohoff FW, Wang Z, Sciortino N, Harper D, Li Y (2009). DAT genotype modulates brain and behavioral responses elicited by cigarette cues. Neuropsychopharmacology.

[49] Franklin TR, Wang Z, Li Y, Suh JJ, Goldman M, Lohoff FW (2011). Dopamine transporter genotype modulation of neural responses to smoking cues: confirmation in a new cohort. Addict Biol.

[50] Wang Z (2012). Improving cerebral blood flow quantification for arterial spin labeled perfusion MRI by removing residual motion artifacts and global signal fluctuations. Magn Reson Imaging.

[51] Field M, Cox WM (2008). Attentional bias in addictive behaviors: a review of its development, causes, and consequences. Drug Alcohol Depend.

[52] Hong LE, Hodgkinson CA, Yang Y, Sampath H, Ross TJ, Buchholz B (2010). A genetically modulated, intrinsic cingulate circuit supports human nicotine addiction. Proc Natl Acad Sci U S A.

[53] Haber SN, Knutson B (2010). The reward circuit: linking primate anatomy and human imaging. Neuropsychopharmacology.

[54] Rogers RD, Ramnani N, Mackay C, Wilson JL, Jezzard P, Carter CS (2004). Distinct portions of anterior cingulate cortex and medial prefrontal cortex are activated by reward processing in separable phases of decision-making cognition. Biol Psychiatry.

[55] Hampton AN, O'Doherty JP (2007). Decoding the neural substrates of reward-related decision making with functional MRI. Proc Natl Acad Sci U S A.

[56] Botvinick MM, Braver TS, Barch DM, Carter CS, Cohen JD (2001). Conflict monitoring and cognitive control. Psychol Rev.

[57] Botvinick MM, Cohen JD, Carter CS (2004). Conflict monitoring and anterior cingulate cortex: an update. Trends Cogn Sci.

[58] Robinson TE, Berridge KC (1993). The neural basis of drug craving: an incentive-sensitization theory of addiction. Brain Res Brain Res Rev.

[59] Schoenmakers TM, de Bruin M, Lux IF, Goertz AG, Van Kerkhof DH, Wiers RW (2010). Clinical effectiveness of attentional bias modification training in abstinent alcoholic patients. Drug Alcohol Depend.

[60] Kong G, Larsen H, Cavallo DA, Becker D, Cousijn J, Salemink E (2015). Re-training automatic action tendencies to approach cigarettes among adolescent smokers: a pilot study. Am J Drug Alcohol Abuse.

[61] Allen SS, Bade T, Hatsukami D, Center B (2008). Craving, withdrawal, and smoking urges on days immediately prior to smoking relapse. Nicotine Tob Res.

[62] Franklin TR, Allen SS (2009). Influence of menstrual cycle phase on smoking cessation treatment outcome: a hypothesis regarding the discordant findings in the literature. Addiction.

[63] Allen SS, Hatsukami DK, Christianson D, Nelson D (1999). Withdrawal and pre-menstrual symptomatology during the menstrual cycle in short-term smoking abstinence: effects of menstrual cycle on smoking abstinence. Nicotine Tob Res.

[64] Cosgrove KP, Mitsis EM, Bois F, Frohlich E, Tamagnan GD, Krantzler E (2007). 123I-5-IA-85380 SPECT imaging of nicotinic acetylcholine receptor availability in nonsmokers: effects of sex and menstrual phase. J Nucl Med.

[65] Harlow SD, Ephross SA (1995). Epidemiology of menstruation and its relevance to women’s health. Epidemiol Rev.

[66] Sinha R (2009). Stress and addiction: a dynamic interplay of genes, environment, and drug intake. Biol Psychiatry.

[67] Perkins KA, Karelitz JL, Giedgowd GE, Conklin CA (2013). Negative mood effects on craving to smoke in women versus men. Addict Behav.

