# Supplementary material for: Influence of menstrual cycle phase on resting-state functional connectivity in naturally cycling, cigarette-dependent women
Source: Biol Sex Differ. 2016 May 10;7:24. doi: 10.1186/s13293-016-0078-6 (PMC4862059; doi:10.1186/s13293-016-0078-6)
Supplement: Additional file 1: — Menstrual Cycle Questionnaire. The menstrual cycle questionnaire (MCQ) used in this study. (DOCX 32 kb) [file 13293_2016_78_MOESM1_ESM.docx]

**SUPPLEMENTAL INFORMATION**

**Influence of menstrual cycle phase on resting-state functional connectivity in naturally cycling, cigarette-dependent women**

Reagan R. Wetherill, Kanchana Jagannathan, Nathan Hager, Melanie Maron, and Teresa R Franklin

**SUPPLEMENTAL CONTENT**

Menstrual Cycle Questionnaire

**Please CIRCLE YOUR ANSWERS to the following questions and PAY CLOSE ATTENTION TO THE DIRECTIONS.**

#### Are you Male or Female?

1= Male 2= Female [If “Male”, DO NOT CONTINUE]

#### Are you at least 1 year post-menopausal?

0= No 2= Yes [If “Yes”, DO NOT CONTINUE]

#### Do you menstruate?

0= No 1= Yes [If “No”, DO NOT CONTINUE]

#### What method of birth control will you be using during the next two months?

**(Circle an answer choice BELOW)**

| **1** = Oral Contraceptives | **2** = IUD | **6** = Diaphragm |
| --- | --- | --- |
| - If so, what kind?  ______________________ | **3** = Condom with spermicidal lubricant | **7** = Hysterectomy (Partial/Full) |
| - How long have you been on an oral contraceptive regularly?  ______________________ | **4** = Approved Hormone Injection of Implant  **5 =** Abstinence (Not engaging in sexual relations) | **8** = Tubal Ligation ("tubes tied")  **9 =** Other: __________________ |
| - When is your “week off?”  _______________________ |  | **10** = No Birth Control |
|  |  |  |

#### Average length of menstrual cycle:

#### 1= 26 2= 27 3= 28 4= 29 5= 30 6= Other: ____

#### 6) Currently, to what degree are you experiencing problems or discomfort associated with your menstrual cycle?

0= None 1= Mild 2= Moderate 3= Severe

#### Currently, are you receiving medical care for any problems or discomfort you may experience related to your menstrual cycle?

0= No 1= Yes If “Yes”, please describe: **_____________________________**

#### First Day of Last Menstrual Flow (first day of last period):

Month / Day / Year

___ ___ / ___ ___ / ___ ___ ___ ___

**CONTINUED ON NEXT PAGE 🡪**

1. **At any time during the 2 weeks prior to getting your period, what is the degree to which you experience any of the following features (Circle the number: 0=None 6=Severe).**

|  | **NONE** | **MILD** | | **MODERATE** | | **SEVERE** | |
| --- | --- | --- | --- | --- | --- | --- | --- |
| Acne | 0 | 1 | 2 | 3 | 4 | 5 | 6 |
| Bloating | 0 | 1 | 2 | 3 | 4 | 5 | 6 |
| Breast tenderness | 0 | 1 | 2 | 3 | 4 | 5 | 6 |
| Dizziness | 0 | 1 | 2 | 3 | 4 | 5 | 6 |
| Fatigue | 0 | 1 | 2 | 3 | 4 | 5 | 6 |
| Headache | 0 | 1 | 2 | 3 | 4 | 5 | 6 |
| Hot flashes | 0 | 1 | 2 | 3 | 4 | 5 | 6 |
| Nausea, diarrhea, constipation | 0 | 1 | 2 | 3 | 4 | 5 | 6 |
| Palpitations | 0 | 1 | 2 | 3 | 4 | 5 | 6 |
| Swellings (hands, ankles, breasts) | 0 | 1 | 2 | 3 | 4 | 5 | 6 |
| Angry outbursts, violent tendencies | 0 | 1 | 2 | 3 | 4 | 5 | 6 |
| Anxiety, tension, nervousness | 0 | 1 | 2 | 3 | 4 | 5 | 6 |
| Confusion, difficulty concentrating | 0 | 1 | 2 | 3 | 4 | 5 | 6 |
| Crying easily | 0 | 1 | 2 | 3 | 4 | 5 | 6 |
| Depression | 0 | 1 | 2 | 3 | 4 | 5 | 6 |
| Food cravings (sweets, salts) | 0 | 1 | 2 | 3 | 4 | 5 | 6 |
| Forgetfulness | 0 | 1 | 2 | 3 | 4 | 5 | 6 |
| Irritability | 0 | 1 | 2 | 3 | 4 | 5 | 6 |
| Increased appetite | 0 | 1 | 2 | 3 | 4 | 5 | 6 |
| Mood swings | 0 | 1 | 2 | 3 | 4 | 5 | 6 |
| Overly sensitive | 0 | 1 | 2 | 3 | 4 | 5 | 6 |
| Overwhelmed | 0 | 1 | 2 | 3 | 4 | 5 | 6 |

#### Have you ever been diagnosed with PMS (Premenstrual Syndrome)?

0= No 1= Yes

#### Have you ever been diagnosed with PMDD (Premenstrual Dysphoric Disorder)?

0= No 1= Yes
